# Feedback on audit and action planning for dental caries control: a qualitative study to investigate the acceptability among interdisciplinary pediatric dental care teams

**DOI:** 10.3389/froh.2023.1195736

**Published:** 2023-06-30

**Authors:** Joana Cunha-Cruz, Juliana Balbinot Hilgert, Catherine Harter, Marilynn L. Rothen, Kim Hort, Elizabeth Mallott

**Affiliations:** ^1^ Department of Oral Health Sciences, School of Dentistry, University of Washington, Seattle, WA, United States; ^2^ Department of Clinical and Community Sciences, School of Dentistry, University of Alabama at Birmingham, Birmingham, AL, United States; ^3^ Post Graduate Program in Epidemiology, Universidade Federal do Rio Grande do Sul, Porto Alegre, Brazil; ^4^ Post Graduate Program in Dentistry, Universidade Federal do Rio Grande do Sul, Porto Alegre, Brazil; ^5^ SouthEast Alaska Regional Health Consortium (SEARHC), Juneau, AK, United States

**Keywords:** health equity, dental caries, qualitative research, audit and feedback, evidence-based dental care, Alaska native

## Abstract

**Introduction:**

American Indian and Alaska Native children suffer from the poorest oral health of all populational groups in the United States. Evidence-based practices (EBP) for caries control are well established, but systematically implementing such practices have proven difficult. Audit and feedback with goal setting, and action planning to implement these EBPs have not been tested or adapted for Alaska Native healthcare settings. The aim of this study was to investigate acceptability and perceived feasibility of an audit and feedback intervention for pediatric dental caries control among dental providers and patient stakeholders.

**Methods:**

The pilot program was implemented in two dental clinics from a tribal healthcare consortium in Alaska. Key-informant interviews were conducted to investigate the contextual, organizational, and behavioral facilitators and barriers to the implementation and expansion of the program. Interview transcripts were analyzed by two researchers using thematic analysis.

**Results:**

Eight key informants were interviewed twice (during and after the intervention period), and one once, for a total of 17 interviews. Patient stakeholders were not interviewed due to COVID-19 pandemic clinic closures and social isolation mandates. Three principal themes emerged: a positive organizational climate and culture fostered the acceptability of the program, the positive impacts of the program observed in the pediatric dental teams and the organization, and the challenges to implement the program including understanding the data reports, trusting the accuracy of the data, and competing priorities.

**Conclusions:**

The intervention of audit and feedback with goal setting and action planning was well accepted and perceived as feasible by the study participants given the financial and human resources provided by the research project. This qualitative study can inform the design and evaluation of process-oriented implementation strategies geared towards decreasing health inequities and improving health outcomes, such as dental caries in American Indian and Alaska Native children and adolescents.

## Introduction

Inequities in oral health persist for low-income, rural and minoritized children and adolescents (1–4). Particularly, American Indian and Alaska Native (AIAN) children suffer from the poorest oral health of all population groups in the United States (1, 4). Evidence-based practices (EBP) and treatments with established effectiveness based on systematic reviews and clinical trials are available to control dental caries (5–8). While certain structural factors (e.g., geographic isolation, shortage of dentists) contribute to the difficulties in disseminating and implementing such practices in Alaska Native settings, dental providers, in general, do not systematically implement office systems to ensure that EBPs are comprehensively used to control dental caries.

Potential office systems strategies selected for such implementation first require rigorous research and testing. Office systems such as implementing polices, disseminating guidelines, providing feedback based on data reports, and goal-oriented processes are strategies that have been shown to increase the adoption of evidence-based practices, reduce healthcare costs and improve patient outcomes (9–12). Although these office-systems have been evaluated and determined as effective as behavior change interventions and policies for primary health care professionals (9–12) and are based on organizational change theory (13, 14), they have not yet been combined and tested for dental caries control nor have they been adapted to Alaska Native settings. The implementation strategies could potentially change the care delivery system in the direction of population-, and evidence-based care delivered by a team of dental providers with timely evaluation to improve oral health and reduce inequities at the individual and population levels. However, the successful implementation of office-systems or implementation strategies should consider the organizational climate within the healthcare setting (15), the experience, and cultural background of providers and recipients of the EBPs and the American Indian/Alaska Native context (16). Chambers and Norton have warned us that “there is ample documentation of mismatches among interventions, the populations they target, the communities they serve, and the service systems where they are delivered” (17). Health interventions that adapted appropriate cultural meaning and context into the intervention materials, messages and delivery systems were more effective than those that did not (18).

In developing a theoretical framework for assessing acceptability, Sekhon et al. (19) defined acceptability as a “multifaceted construct that reflects the extent to which people delivering or receiving a healthcare intervention consider it to be appropriate, based on anticipated or experienced cognitive and emotional responses to the intervention.” Particularly for pilot feasibility studies, acceptability can play a key role in identifying necessary changes required to ensure that the intervention and the future large-scale study are acceptable and feasible (19). Our specific aim in this study was to investigate the acceptability and perceived feasibility to office system strategies used in a caries control pilot program among dental providers and patient stakeholders.

## Material and methods

### Study design

This descriptive qualitative study conforms to the Standards for Reporting Qualitative Research (20). This study design was utilized to enable an in-depth exploration and assessment of acceptability and perceived feasibility of a pilot program, including the contextual, organizational, and behavioral facilitators and barriers that could inform changes to the program for a larger implementation study. This study received ethics approval from the Institutional Review Boards of the Alaska Area and the University of Washington. A consent form describing in detail the study procedures and risks were given to the participants prior to data collection and informed consent was obtained from participants.

### Study setting

The setting was the dental department at a large health care organization in Alaska; a non-profit health consortium of 18 Native communities of Southeast Alaska. The organization provides medical, behavioral, and dental care to adults and approximately 7,000 children and adolescents (insured, sliding fee-for-service and uninsured Native and non-Native). The implementation sites of the program were the dental clinics in Haines and Kake. The community of Haines has a population of almost 2,000 with most residents identifying as White (78%) and Alaska Native or American Indian (11%). The resident child and adolescent population (age 0–22) was 323 in 2019. Kake has a smaller population of almost 600 and most residents identify as Alaska Native or American Indian (72%). The child and adolescent population was 138 in 2019.

### Intervention

The Oral Health Equity for Alaska (OHEAL) pilot program implemented an evidence-based, and theory-informed program to disseminate EBPs in Alaska Native settings to control dental caries. OHEAL was an intervention that consisted of two mutually reinforcing implementation strategies at the organization and the clinical practice levels: 1) office practices: feedback on audit, goal setting and action planning, and 2) office tools that fit the practice, its patients, and local conditions: EBP guideline on dental caries manual, data reports, and feedback and action plan manual. These components, along with insight into cultural-specific attitudes, values and context of the patient population, worked together to create an intervention to foster the consistent use of EBPs for dental caries control to all pediatric patients.

In OHEAL, dental providers (pediatric dentists, general dentists, dental therapists, and dental health aides) worked as a team to provide annual caries assessment and tailored dental care to children and adolescents in the communities. The teams adapted the tools, created goals for care delivery and their performance was monitored to help them meet those goals. Workshops with members of the tribal organization were used to adapt EBP guidelines on caries control and office-systems tools and strategies prior to implementation in the two communities. During the workshops, participants identified barriers and facilitators for implementing the guidelines, and improving the oral health of the communities being served. They also identified goals and benchmarks for the audit reports and suggested changes to the text and graphics presented in the audit reports, and manuals. The audit report displayed graphically and textually data related to the four goals selected by the members with a visual benchmark. Data for all clinics were anonymously presented in the graphics, except for the specific clinic that the report pertained to, which was identified. This allowed providers and administrators to compare their performance in relation to other clinics. Dental providers were then supported by facilitators within their own organization who monitored their performance, helped them identify barriers to achieving goals and to collaboratively find solutions and plan for action. These evidence-based (9–12) and organizational theory-informed (13, 14) behavioral change intervention strategies and tools were novel in this setting.

### Sampling strategy

Purposeful sampling was utilized to identify and select participants. Individuals met inclusion criteria if they: spoke English, were currently a full- or part-time employee of the tribal organization and had direct or indirect involvement in the implementation of OHEAL. Originally, we had planned to include patients (caregivers) and community stakeholders, but limitations in travel and clinic closures due to the COVID-19 pandemic made this impossible. The research team identified potential eligible participants and they were first approached by the head of the pediatric dental program *via* email invitation, then by the research coordinator (CH) *via* phone call.

### Data collection

Data were collected through semi-structured key informant interviews at mid-intervention in September and October 2019 and post-intervention between May and July 2020. Following recommendations from the Theoretical Framework for Acceptability (19), data were collected at two time points during the research project. The findings from the two periods were reported in combination because of the similarity of their content. The interview data reached saturation, in that all organizational leaders and dental providers involved in the project were interviewed, and the same themes emerged repeatedly, with no new information being provided (21). The interviewer (CH), trained in qualitative research methods, conducted all the interviews using a guide with open-ended questions developed by the investigators to elicit participants’ perceptions of the current practices and organizational systems in the dental department of the tribal care organization and then specific questions regarding the OHEAL strategies and tools. The guides for the mid- and post-intervention interviews contained the same prompts, except for the inclusion of a prompt about leadership support in the post-intervention interviews (see Appendix).

The Theoretical Domains Framework (TDF) offered the basis in which to explore cognitive and emotional responses to change processes (22) and it has been applied in exploratory, formative studies, such as this, to help elicit participants' perceptions of a given intervention strategy (23). Of the 14 TDF domains, the interview guide included questions pertaining to 8 domains: knowledge, skills, social/professional role and identify, beliefs about capabilities, goals, environmental context and resources, social influences, and behavioral regulation (23).

### Data analysis and processing

The interviewer (CH) provided a study informational sheet for participants to review at least two days prior to the interview and verbal consent and authorization to record was obtained prior to the start of the interviews. Mid-intervention interviews were conducted by phone and audio recorded. Post-intervention interviews took place *via* Zoom (audio only) and were recorded. Once the interview was complete, the interviewer (CH) downloaded recordings on a password protected laptop into an encrypted folder with only the date of the interview. Audio recordings were transcribed, reviewed for accuracy by the investigators (CH) and the transcripts uploaded into Dedoose Version 8.3.17 (Sociocultural Research Consultants, Los Angeles, CA). The recordings were subsequently deleted from the recording device. No identifiable information was collected for or connected to the interview recordings or the transcripts, demographic information was collected separately.

Interview transcripts were analyzed by two researchers using inductive-deductive thematic analysis (24, 25). An initial codebook was developed *a priori*, based on the research questions and the theoretical framework, and then, inductive codes were assigned to segments of data that described a new theme observed in the text during coding. The transcripts were analyzed in an iterative process by the coders (CH and JH) by first separately reading the raw data to familiarize with it, identifying key ideas and patterns, and labeling these ideas with codes. After reading and independently applying primary and secondary codes for each transcript and adding any new emergent codes to the existing codebook, the coders formulated these codes into broader categories. Next, they discussed codes that were missed or disagreed upon until they reached 100% agreement. Using Dedoose software, the codes were digitally marked in the transcripts by one coder and then compiled to create code reports with associated data and quotes. The reports were reviewed and synthesized further into broader theme domains and subdomains by both coders, forming the basis of our findings.

## Results

Nine participants were interviewed mid-intervention and eight were interviewed for post-intervention for a total of 17 interviews. Participants' age ranged from 26 to 45 years old, 77% were female and 33% were Alaska Native or American Indian. Interviewees were dental providers, and their degrees/specialty were dentist, pediatric dentist, dental hygienist, dental therapist, and dental health aide. Four participants had additional administrative roles as clinic directors or manager. One participant was lost after the mid-intervention interview due to leaving the job at the organization. Interviews lasted from 30 to 70 min.

Our data revealed three major themes: (a) the culture and climate of the tribal care organization may have encouraged the acceptability of OHEAL; (b) OHEAL brought about positive changes and new insights for the dental department as a whole; and (c) OHEAL had implementation challenges and competing priorities within the department. These themes were related to the TDF domains mapped in Table 1.

**Table 1:** Relationship between the emergent themes and the TDF domains.

Interview theme	TDF Domains
Organizational Climate and Culture of the tribal care organization	Social/Professional role and identify Environmental context and resources Social influences
Positive changes and insights from the OHEAL intervention	Knowledge and Skills Social/Professional role and identity Beliefs about capabilities Goals Social influences Behavior regulation
Challenges and Opportunities for improvement of the OHEAL intervention	Knowledge Social/Professional role and identity, Beliefs about capabilities Environmental context and resources

### Organizational climate and culture of the tribal care organization

All participants described the organizational culture and climate in the dental department positively, as having engaging staff dedicated to the organization's mission and to the communities it serves. They described activities such as working during weekends and lunch hours to illustrate this dedication. Others described team members as flexible, open-minded, and willing to build trust and respect with the AIAN community and culture. Additionally, others described the organization and dental clinic as trailblazing and cutting edge, specifically for their pediatric population. As one participant noted,

*“The fact that our clinic has a children's clinic, and we have pediatric dentists on staff is already tremendously different than what other tribal programs have in terms of staffing and dental services. I think that by the nature of our organization's emphasis on staffing and space for kids, access to [dental] care is greater than it is at other programs [from other dental care organizations].”* (Participant A)

Although participants felt positively about the organization and its willingness to incorporate change and openness to innovative ideas, there was hesitation related to organizational resources to expand and sustain the OHEAL program beyond the scope of the current research project, ie, a research project with a defined short intervention period, in a small geographical area (two communities). Resources cited as needed to run the program across the organization sites included the funds and logistics to add staff support with the skills to extract data from the digital dental practice management software and produce accurate data reports, to add non-clinical administrative time for providers to discuss the data reports and create an action plan, and to then implement the plan which involved flying in dentists and dental therapists to the different communities. One participant explained,

*“I need to make sure I have staff to do that at all of those sites, which is a priority as well. So, thinking about what it takes to provide care in terms of staff and supplies, we can't achieve those goals without the staff and supplies.” (Participant D)*

They did, however, feel they had sufficient clinical training, emotional intelligence, tools for communication, and a shared understanding of the mission and goals of the organization that facilitated the implementation of the OHEAL intervention.

### Positive changes and insights from OHEAL

Participants described many positive changes and new insights OHEAL brought to the organization and dental department specifically in the communities in which it was implemented. Overall, participants appreciated the emphasis the program gave to regular and consistent communication with team members. The topics of the regular and consistent communications among team members were also noted as novel and important, such as evaluation of ongoing projects and ideas, standardized training, accountability, setting achievable goals, reviving community partnerships, and decreasing operating room visits by emphasizing prevention and non-surgical caries control. One participant said,

“*OHEAL just came in and brought the light into the room. Those [meetings, reports, and communications] have been all new ways of approaching the same problem, and we got this renewed enthusiasm from being part of the OHEAL program*” (Participant B)

After receiving the data reports summarizing goal status over a year, participants were able to keep track of goal achievement with actual data, not just anecdotes. They believed these reports created more accountability and transparency. For all participants, this was the first time they visualized and tracked their goal status over time and appreciated the frequency of how often the reports were delivered (once every two months). According to participants, these reports helped guide meetings, were (eventually) easy to interpret, and were effective in describing treatment differences between age groups and across clinics. One participant noted,

“*It's nice to get that information. You could see where you're doing a great job and what you're missing. It*”*s pretty eye opening, and it makes people more willing to make those phone calls and track down moms.”* (Participant C)

According to about half of the participants, feedback on audit meetings that were set in place were seen as a great reminder to check in, problem solve and action plan. The participants noted the meetings were structured in a way that team leaders scheduled and lead the meetings and met with each provider from each community working towards the OHEAL specific goals (typically, not in person, but by phone or video conference).

“*What's been helpful [about OHEAL] is that accountability piece for regular meetings, because we're able to talk about things when they come up rather than waiting for them to become big things that are harder to solve. To talk about little things that are going on, that's helpful in terms of problem solving. But in terms of getting a project from start to finish, it requires us to regularly be working on things.” (Participant A)*

Since the program was implemented, participants described some patient-specific benefits they observed. Some noted more appointments available due to providers traveling out to communities more often, creating more opportunities for patients to be seen, and adjusting the way they schedule these visits, shortening time to treatment for children with active decay.

“*I noticed a huge improvement on community access. Kids getting in more frequently than before, because during this project it was identified that there is only one provider traveling out to the community right now. And we need to give them support. And identifying where it is that support needs to come from. That was a big thing that came from this project.” (Participant E)*

As a result of barriers identified during the feedback meetings, an action plan implemented was the training of dental health aide therapists in more preventive and conservative restorative clinical procedures such as silver diamine fluoride applications and Hall crowns, allowing them to work at the top of their scope of practice and effectively see more pediatric patients. By performing these less invasive techniques, it translated into keeping providers as “regulars” in the remote communities, building additional trust with the communities. Others explained how, to overcome barriers identified during the feedback meetings, they established chart reviews, and a pediatric patient panel to track patient visits. In addition, they adjusted their schedules to allow for more pediatric patients and worked with the medical clinic as well as social media to connect with children who were not being seen in the dental clinic. One participant described that they felt more engaged and accountable, changing the way they facilitate and manage team members.

“*What changed wasn't the way I practiced clinically, but the way I facilitated program management. Having data to utilize and having conversations in the format that we developed was new and different and certainly driven by the data and the project.” (Participant A)*

Participants expanded on what insights they gained by working on the OHEAL project and others described changes they have made as a result. For example, participants discussed the inability of their current dental practice management software to straightforwardly produce effective and meaningful data reports. This led to a larger conversation regarding the lack of transparency in goal status and achievement within the department. During these interviews, suggestions arose on how to address these concerns by scheduling more regular, structured, department-wide meetings, making goals more visible to the whole department, adjusting goals as they become easier to attain, and evaluating the data at the provider level in addition to the clinic level.

“*If we”re setting goals, each site needs to get site specific data, so they know exactly where they’re at and how they compare to everybody else. Then identify what the barriers are for their own site and identify what they should be doing. They could pick a goal or two; work on those and get feedback. We could all benefit from each other*’*s experiences.” (Participant E)*

### Challenges and opportunities for improvement

As participants noted, implementation was not without its challenges. Many participants explained specific challenges with the tools and the feedback on audit and action planning meetings while others described more general opportunities for improvement. One concern from participants who were not involved in the day-to-day processes of the intervention was just how accurate were the data reports, or how the non-clinical time of providers were being used. By contrast, those who were directly involved wanted to find a way to show others in the organization the value of the tools and meetings.

Specific concerns voiced about the data reports and action planning tool included the lack of aggregate data of each report they received over the implementation period and the inability to easily track and combine action plans to refer to and check their progress. Additionally, participants reported that goal monitoring with the provided data reports made some participants question the accuracy of the data collected due to classification systems and variables potentially misrepresenting the community data. As one participant expressed,

“*Does this number mean what I think it means? It's saying something completely different; we thought we were being more successful by getting patients on a regular protocol. But our actual success wasn't what we thought it was, or maybe we're measuring wrong. Maybe we just had too many patients and it hadn't been a year yet.” (Participant F)*

Lastly, participants suggested adjustments to how and with whom meetings were scheduled. Specifically, a participant noted that scheduling meetings with both OHEAL teams from the two communities that participated in the intervention to discuss and action plan together, instead of having separate meetings, would increase transparency, communication and help others understand the status of goals in other clinics.

## Discussion

This study provides evidence regarding the feasibility and acceptability of an evidence-based, adapted implementation effort to improve oral health and eliminate AIAN inequities. The program consisted of a way to evaluate and act on solutions needed to achieve organizational goals for pediatric oral health. Most participants would recommend this program to other clinics (within the organization and beyond) and have identified positive aspects of implementing the program, the challenges associated, and potential improvements to the program for future interventions.

Positive aspects of the process of implementing the program included the supportive organizational climate and culture, the incorporation of communication, goal setting and action planning, and reinforcing professional roles with improvement of skills and beliefs about capabilities. The organizational climate and culture were reported by the participants as not only a facilitator to implementation but also may reinforce the acceptability of a program such as OHEAL. These results are consistent with the positive organizational climate observed in a recent survey done with this dental organization (26). This finding relates to the TDF domains social/professional role and identity, environmental context and resources, and social influence, which are seen as organizational factors as opposed to individual level factors that may lead to successful behavior change (27). In an organization, the management level of a workplace communicates values to its team regarding policies, practices, and processes (28). In addition to organizational climate and culture, implementation climate (29) is “a shared perception that innovation use is expected, supported, and rewarded.” This supports the data provided in this study, in suggesting that without specific characteristics such as professionalism, organizational commitment, and ethical climate (30), an intervention may not be deemed acceptable by staff responsible for implementation (31–33).

Positive changes because of the OHEAL program included incorporating clear and consistent communication, systematically keeping track of goal progress and achievement, and instilling action planning and accountability. Communication plays a key role in successful implementation efforts: it includes more than simply the transference of information from one party to the next, but requires a shared understanding between parties and focuses on changes because of that communication (34). According to participants, implementing OHEAL opened lines of communication and collaboration to reach common goals. It also gave the care team the opportunity to self-set goals for this intervention. This process is supported by the literature in that self-set goals may be more enticing, motivating, and feel more achievable than organizational high-level goals (35–38). An additional aspect of the intervention was the quantifiable nature of the data reports showing progress on these goals. Seeing and discussing the progress on a regular basis made participants feel they had a system of accountability and reaffirms the TDF domains regarding goals, beliefs about capabilities, and behavior regulation. So not only are participants self-setting goals, they are more accurately self-assessing their performance through audit and feedback (39), thus creating intentions to change behavior and improve practice (40). Audit and feedback paired with an action plan (in this intervention through data monitoring and action planning) give a more focused strategy to utilize time and resources towards more efficient improvements and solutions (41, 42).

Another positive change of the OHEAL program was the reinforcement of the professional role and identity of dental therapists as dental providers, with increased skills and belief in their capabilities in pediatric caries control. A new insight from this study was that having the dental therapists present was not what is perceived to improve care to pediatric patients; it was the training and autonomy of the dental therapists to work at the top of their scope of practice to provide both preventive and restorative treatments to the AIAN pediatric population. Many research studies have already shown benefits of incorporating dental health aide therapists into the dental care team in AIAN communities (34, 43–45). Dental therapists and dentists were trained and calibrated for assessing caries risk, and following a caries control protocol for timely prevention and treatment, thus expanding care capabilities and enabling better efficiency during appointments.

With positive changes came specific challenges. Providers and administrators identified some aspects of the program that difficulted its implementation. Participants who were not directly involved in the day-to-day implementation of OHEAL were not convinced of the effectiveness of non-clinical time usage for discussing and evaluating goals, reading data reports, and action planning. This intervention brought about a true concern for organizations that depend on the financial productivity of clinical appointments and treatment: is taking the time to audit, provide feedback and plan for action worth the time commitment away from clinical care and potential fiscal loss. Additionally, when interpreting and discussing the data reports, some participants felt they still required more training to fully achieve the benefit of reviewing these reports. According to the American Dental Education Association, dental and dental hygiene students' curriculum includes epidemiology, but it seems limited and students do not delve into data interpretation (46). The same might be true for dental therapists (47). Knowing this, any future intervention including data reports or data monitoring for dental teams will most likely require appropriate buy-in from leadership for non-clinical time usage and training and support from those with formal training in epidemiology or data science.

As a result of this qualitative study, we have identified aspects of this intervention that would benefit from improvements. This study suggests that more visibility and transparency within the dental department on how one clinic's performance compares to others in the consortium could be beneficial for providers. These results align with the literature suggesting that seeing other's goal progress may positively affect motivation to achieve goals (37, 38). Some participants summarized a more effective communication strategy including more frequent all-staff department meetings with structured agendas for consistency and a central data repository of guidelines, standard operating procedures, and clinical audit reports to refer to for transparency and standardization of care within the organization. Future interventions may include the entire organization as opposed to just two communities within the department as the separate goals for the intervention and the organization may have created competing priorities for participants.

Even though we had a good representation of the practitioners and administrators involved with the intervention (i.e., good internal validity), because this was a pilot program, the number of persons interviewed for this qualitative study was small and thus our findings should not be applied to other populations or contexts. In addition to the limited generalizability, this study is limited by our inability to include patient, caregivers, and community members' perspectives on the OHEAL program. We had planned to include these stakeholders, but limitations in travel and clinic closures due to the COVID-19 pandemic made this impossible, thus missing a key perspective and data that would benefit future research. Lastly, self-reported data from participants associated with the organization may result in social desirability bias. The results are mostly positive with specific challenges and recommended improvements noted. All pariticpants are employees of the organization and views may skew positively as they want their work and workplace to be successful. While we cannot fully control for this bias, we utilized open-ended and non-leading questions in our interviews (see Appendix). In addition, we assured participants that information shared during the interviews were kept confidential, and no identifiable information was stored with the interview data transcripts.

When engaging in self-reflection during the conduct of this research, the authors were aware that their positionality may have influenced the findings. Three co-authors are Latina, non-Indigenous, persons, and one is a white, non-Indigenous person and they have no personal experience with the challenges faced by AIAN children and families. Two co-authors are white, non-Indigenous persons, with professional experience with the challenges of providing care to AIAN children and families. In common among us are the commitment to conducting research that is respectful of the Alaska Native individuals and communities. We acknowledge the potential of our own biases and assumptions when collecting, interpreting, and reporting the data from this study.

## Conclusion

Given that evidence-based practice guidelines and protocols for dental caries control are available but are not used systematically and widely (48–50), this formative work testing goal setting, data audit and feedback and action planning indicated that it is acceptable and challenging to implement new office systems in dentistry. Currently, there is very scarce literature on the effect of non-clinical tasks and office-systems in dental care practices and oral health outcomes. This qualitative study can inform the design and evaluation of large-scale multisite studies on dissemination and implementation science, and more specifically, audit and feedback, for the use of evidence-based practices for dental caries control aiming to eliminate inequities in oral health, particularly for American Indian and Alaska Native children and adolescents. This study is distinct in its use of the TDF to guide data collection and develop an understanding of how the domains apply to the experiences and perceptions of those involved in this implementation. The implementation strategies were perceived to be acceptable and feasible, and the study highlighted the changes needed to implement the study more widely within the dental care organization or across organizations.

## Data Availability

The datasets presented in this article are not readily available because tribal authorizations for public data access were not obtained by the research team. Requests to access the datasets should be directed to: Joana Cunha-Cruz, JoanaCCruz@uab.edu.
