# Supplementary material for: Feedback on audit and action planning for dental caries control: a qualitative study to investigate the acceptability among interdisciplinary pediatric dental care teams
Source: Front Oral Health. 2023 Jun 30;4:1195736. doi: 10.3389/froh.2023.1195736 (PMC10348878; doi:10.3389/froh.2023.1195736)
Supplement: Supplementary file 1 [file Datasheet1.pdf]

Interview Guide for Organization Interviews with SEARHC Staff

This guide is provided for the interviewer to use during the interviews. The questions will be slightly adapted depending on whether the interviewee is a healthcare professional or an organization implementation leader.

Stage I: Introduction and informed consent process (5 minutes)

Thank you for meeting with me today. I am________________. I work in the dental school at the University of Washington in Seattle.

I may take a few notes, but they will mostly be to help me keep track of topics. I would like to record our interview because I cannot take notes fast enough to get everything you say, and don't want to miss anything. We will not share the recording with anyone other than the study team. Is that OK with you?

. The purpose of our interview today is for me to hear your thoughts on the OHEAL project and what you liked and didn't like about it. What you tell me today will be shared with SEARHC to improve dental care for children and teens. Your personal information will not be attached to what we share.

In our reports, we will summarize what we have heard from everyone we talk with. If we use any quotes from what you tell me, those quotes will not be linked to you.

I have sent you an informational sheet that explains this interview process.

Do I have your consent to continue with this interview?

Do you have any questions for me before we get started?

Stage II: Questions and Confirmation

As a bit of an introduction to the questions: We are worked with SEARHC to make some changes in how the organization provides dental care for children and teens. We're gathering opinions on how that's going from lots of different folks in SEARHC. Today, we will be talking about activities that some of the SEARHC staff have been doing at a few of the village clinics.

Before we get started, I have a few questions about you.

What is your age?

- [ ] 16-17
- [ ] 18-20
- [ ] 21-25
- [ ] 26-35
- [ ] 36-45
- [ ] 46-55
- [ ] 56-65
- [ ] 66-75
- [ ] 76 or older

What is your gender?

- [ ] Male
- [ ] Female
- [ ] Other ___________

Are you Hispanic or Latino?

- [ ] Yes
- [ ] No

Do you consider yourself to be… (choose all that apply)

- [ ] White or Caucasian
- [ ] Black or African American
- [ ] American Indian

  Please specify ___________________
- [ ] Alaska Native

  Please specify ___________________

  Are you a beneficiary? [ ] Yes [ ] No
- [ ] Native Hawaiian or Pacific Islander
- [ ] Asian
- [ ] Other: ____

Where do you live?

____

How long have you lived in ______ [this village]?

- [ ] Less than 3 years
- [ ] 3-6 years
- [ ] More than 6 years

What is your role at SEARHC?

(if not mentioned) Which clinical specialty?

Which SEARHC clinic(s) do you work?

____

How long have you been working at SEARHC clinics?
Any SEARHC clinic _______

Kake _______

Haines _________

I have interviewed you before and some of the questions are quite similar, but since this is our final evaluation of the changes, we need to ask them again. There is no problem if you want to skip some questions or if you feel you have answered the question already and there were no changes.

1. Sometimes a clinic or organization sets goals for improving the delivery of care. What goals have been set in ____[village] since April 2019? [*prompt, if needed:* either by SEARHC or others]

2. During the last year did you receive any updated or new clinical care guidelines or recommendations? [*Prompt, if needed*: from SEARHC or others] *[It's ok if they do not receive, just go to next question]*

    - How often did you get them? Where do they come from?
    - How helpful were they? What made them helpful?
    - What would you do to improve the guidelines or recommendations you received? Here, I'm not wondering about the specific guidelines or recommendations, per se, but more about the process by which they are communicated or delivered to you.

As part of the OHEAL project in particular, SEARHC set specific goals in order to improve clinic care for kids and teens. For example, some of these goals included assessing caries risk at the time of the exams, treating active decay as soon as possible, and reaching out to all kids and teens in the community. Are you aware of these specific goals? [*If not aware, ask the following questions about any other goal they are aware of]*

3. Last April (2019), UW researchers did a workshop with some providers from SEARHC where they looked at the data related to the goals and discussed actions that might help achieve those goals. Did you participate?

    - [If yes], how helpful was this experience? What would you do to improve it?
    - [If no but they know about it] How helpful would it be to you to participate in the future?

4. Between April 2019 and now, how did you and your colleagues/team monitor progress in

reaching the goals in ____[village]?

- *Prompt*: "Receiving data reports and talking about changes with Kim or Liz?"
- *Prompt*: "Did you feel as though you were properly trained or prepared for engaging these new activities? Was it fairly new or out of your comfort zone?"
- *Prompt*: "Did you find these activities are helpful or useful?"

I would like to hear more about these activities. Let's talk first about meetings and communications. Did you meet with others to talk about the goals and to monitor or track progress?

- How often did you get [type of communication]?
- How helpful were these communications? [Prompt: Tell me more… or give me one example]
- What would you do to improve the [type of communication]?

5. Next, I want to hear more about the data reports. Did you get any feedback or data reports about your work?

    - How helpful were those reports? What makes them helpful? [Prompt: Tell me more… or give me one example]
    - What would you do to improve the data reports?

6. What have you noticed at the clinic that might be different this past year compared to earlier years?

    - Do you think those differences make the care better or worse? How so?
    - Do you think some of these changes had to do with the monitoring activities?
    - What adjustments to job roles or duties have happened in the past year?
    - What differences make it easier for patients to get care? How so? What differences make it harder? How so?

7. Can you talk about some of the changes you made after seeing the data reports to achieve the goals?

    - *Prompt*: Do you think any of these changes were related to the data reports and monitoring activities?

8. What resources do you think were needed specifically for you in your job to reach the goals for caries assessment and treatment of kids and teens in _______[village]?

    - Do you feel as though there were adequate resources whether in your village/clinic or SEARHC overall to reach these goals and continue to do so overtime after OHEAL?

9. If you could change just one or two things about tracking your progress, what would you change? Why?

10. Would you recommend the monitoring progress activities of data reports and meetings to other dental clinics as a way to change clinic practices? Why or why not?
    - *Prompt*: Do you feel as though these activities had a positive or negative influence on the care and treatment given at SEARHC for children and youth <21 years old?
11. What did you like or dislike about the monitoring activities you've participated in?
12. Did you feel as though you were properly supported by supervisors or administrators you worked with while incorporating these monitoring activities into your workflow?
    - This can include continuous feedback on progress, instruction as needed, emotional support, proper training, regular and timely communication, etc.

You've shared a lot of valuable information with me – thank you for your detailed and helpful answers. Before we end, I'm wondering what else you think we should know about SEARCH and the topics we discussed today.

Thank you very much, again, for sharing your time and thoughts with me today!

____
